# Dana Cole, Georgia Division of Public Health, Notifiable Disease Section, Department of Human Resources, 2 Peachtree Free-living Canada Geese and Antimicrobial Resistance

**DOI:** 10.3201/eid1106.040717

**Published:** 2005-06

**Authors:** Dana Cole, David J.V. Drum, David E. Stallknecht, David G. White, Margie D. Lee, Sherry Ayers, Mark Sobsey, John J. Maurer

**Affiliations:** * Georgia Division of Public Health, Atlanta, Georgia, USA;; † University of Georgia, Athens, Georgia, USA;; ‡ US Food and Drug Administration, Laurel, Maryland, USA;; § University of North Carolina, Chapel Hill, North Carolina, USA

**Keywords:** escherichia coli, antimicrobial drug resistance, environmental microbiology

## Abstract

We describe antimicrobial resistance among *Escherichia coli* isolated from free-living Canada Geese in Georgia and North Carolina (USA). Resistance patterns are compared to those reported by the National Antimicrobial Resistance Monitoring System. Canada Geese may be vectors of antimicrobial resistance and resistance genes in agricultural environments.

The epidemiology of zoonotic diseases is growing in scope and importance in public health as the interface between animal and human habitats narrows and new diseases emerge. Historically, zoonotic disease research has emphasized occupational or animal-origin foodborne exposures. However, environmental exposure pathways to zoonotic pathogens are increasingly documented as foodborne disease surveillance and control efforts prove successful. Nonanimal-origin sources of zoonotic infection, such as raw fruits and vegetables, nuts, and water, are reported more often (1–3). Although fecal contamination of raw food products in fields is an important source of zoonotic infection (1), the source of contamination is usually not determined. Consequently, environmental reservoirs of microbes of public health importance need to be investigated.

Canada Geese (*Branta canadensis*) (Figure) populations have steadily increased in the past 50 years and have become a nuisance in some areas (4,5). The large amount of feces produced by geese congregating around surface water bodies is a source of environmental contamination and, potentially, zoonotic pathogens (4–7). Feces from large flocks are major contributors to fecal coliform levels in reservoirs that supply drinking water for some cities (5,6), and free-living bird populations can serve as reservoirs for pathogenic bacteria such as *Salmonella* (8,9), *Escherichia coli* (10), *Campylobacter* (10,11), *Listeria* (11), and *Chlamydia* (9). Thus, wild bird populations can amplify and eventually transmit infectious microbes to humans by directly contaminating agricultural fields or surface waters used for drinking, recreation, or crop irrigation.

**Figure:**
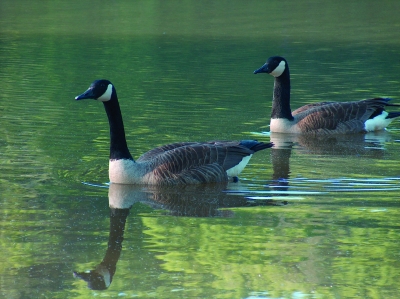
Free-living populations of Canada Geese (*Branta canadensis*) can serve as reservoirs of antimicrobial-resistant bacteria such as *Escherichia coli*..

Free-living and domestic bird populations can also be reservoirs of drug-resistant bacterial pathogens or resistant genetic elements. Antimicrobial-resistant organisms in domestic animals such as poultry, beef, and swine are well documented (12,13) and have been implicated as reservoirs for multidrug-resistant foodborne pathogens. Interaction with waste materials from these livestock species may confer resistant pathogens and genetic elements to free-ranging wildlife, potentially creating an additional environmental reservoir of resistant organisms (8). We examine the impact of habitat on antimicrobial susceptibilities of *E. coli* isolates recovered from different flocks of resident, free-living Canada Geese to determine the potential for these animals to be additional sources of antimicrobial resistance through exposure pathways that originate in the environment.

## The Study

Collaborators from separate regions collected cloacal swabs or fresh guano from Canada Geese at 4 geographically diverse surface water bodies in Georgia (n = 72) and North Carolina (n = 90). Cloacal swabs were taken from each of 24 Canada Geese captured at each Georgia site. At the North Carolina site, groups of resident geese were followed by a study investigator on 8 occasions, and fresh guano was collected from 7 to 8 birds. Geographic locations represented the following land uses: recreational (Stone Mountain Park, GA), agricultural (Craven County, NC; Griffin, GA), and industrial (Lake Juliette, GA). The Craven County site was near a swine housing facility, and geese were observed using swine waste lagoons and adjacent surface waters and farm fields. The Griffin site was not near food animal facilities but was adjacent to test plots used by crops and soils scientists at the University of Georgia Experiment Station. Neither the Griffin Lake nor the lake at Stone Mountain Park is downstream from a sewage treatment plant; however, Lake Juliette, a reservoir for an electrical generation plant, is formed by Rum Creek and water pumped from the Ocmulgee River, on which several sewage treatment facilities reside.

Isolation and biochemical identification of *E. coli* from free-ranging geese were performed as follows. In Georgia, cloacal swabs were transported to the investigators' laboratory and stored at 4°C. The following day, swabs were used to inoculate brain heart infusion broth (BHIB) and incubated overnight at 37°C. BHIB cultures were subsequently streaked for isolated colonies on MacConkey agar plates; 1 lactose-fermenting colony was selected from each goose sample that exhibited growth on agar. In North Carolina, bird samples were pooled and transported to the laboratory on ice in sterile, plastic bottles and stored at 4°C overnight. A 1-g sample of guano was diluted in sterile phosphate-buffered saline, and 3 separate dilutions were filtered through 47-mm, 0.45-μm pore-size, gridded cellulose ester filters. After overnight culture on mFC agar plates at 37°C, filters containing a countable number of fecal coliform colonies (20–100 CFUs) were transferred to plates containing nutrient agar and 4-methylumbelliferyl-β-D-glucuronide (MUG) and incubated for an additional 3–4 h. Colonies fluorescing blue under long wavelength UV light were selected for biochemical confirmation. Up to 5 presumptive *E. coli* colonies were selected from each sampling round. All isolates were identified to genus and species level with the Vitek System (bioMériux Vitek, Hazelwood, MO, USA).

Antimicrobial-susceptibility patterns for each confirmed bacterial isolate were determined by broth microdilution with the Sensititre automated antimicrobial susceptibility system (Trek Diagnostic Systems, Westlake, OH, USA) and interpreted according to NCCLS criteria for dilution susceptibility testing methods when applicable (14). Antimicrobial susceptibilities were assessed for amikacin, amoxicillin/clavulanic acid, ampicillin, apramycin, cefoxitin, ceftiofur, ceftriaxone, cephalothin, chloramphenicol, ciprofloxacin, gentamicin, imipenem, kanamycin, nalidixic acid, streptomycin, sulfamethoxazole, tetracycline, and trimethoprim/sulfamethoxazole. *E. coli* ATCC 25922, *E. coli* ATCC 35218, and *Pseudomonas aeruginosa* ATCC 27853 were quality-control organisms. Differences in the proportion of resistant isolates were analyzed by chi-square test (SAS, ver. 8.01, SAS Institute Inc., Cary, NC, USA).

Isolates were also screened for class 1 integrase gene *intI*1 and integron-associated antimicrobial resistance genes *sul1* and *aadA1* by polymerase chain reaction (PCR). Isolates that exhibited resistance to β-lactam antimicrobial agents were screened by PCR for TEM β-lactamase gene, *bla*_TEM_, with appropriate positive and negative control strains. DNA template for PCR was prepared as described by Hilton et al. (15).

The Table describes antimicrobial resistance phenotypes and associated resistance determinants in *E. coli* isolates recovered from Canada Geese stratified by geographic site. No gram-negative enteric bacteria were isolated from geese at Stone Mountain Park, although *E. coli* isolates were recovered from the bird waste from Griffin, Lake Juliette, and Craven County. The number of isolates recovered was much higher among geese in agricultural areas compared to other land usages (e.g., industrial or recreational). The proportion of isolates resistant to antimicrobial agents was significantly greater (p = 0.0004) among *E. coli* isolates from Craven County geese, where interaction with swine waste lagoons was observed. Antimicrobial resistance patterns in this population matched those most commonly reported for swine *Enterobacteriaceae* from the National Antimicrobial Resistance Monitoring System (NARMS) studies (e.g., tetracycline, streptomycin, sulfamethoxazole, and ampicillin resistance) (12,13). Most *E. coli* isolates (72%) recovered from Craven County geese exhibited resistance to ≥1 antimicrobial agent. In contrast, resistant *E. coli* recovered from agricultural geese in Georgia (Griffin) with no apparent contact with livestock wastes had a lower proportion of resistance (19%) and only exhibited resistance to β-lactam antimicrobial agents (cefoxitin-amoxicillin/clavulanic acid-cephalothin).

**Table:** Antimicrobial resistance phenotypes and genotypes of *Escherichia coli* isolated from Canada Geese sampled in Georgia and North Carolina

Antimicrobial resistance phenotype* and genotype	Site†			
	Lake Julliette	Craven County	Griffin	Stone Mountain
	n = 2	n = 25 (%)	n = 21 (%)	n = 0
Pansusceptible‡	2	7 (28)	17 (81)	
Ampicillin		5 (20)	1 (5)	
Amoxicillin/clavulanic acid		0	2 (10)	
Cefoxitin		0	2 (10)	
Cephalothin		1 (4)	4 (19)	
Tetracycline		16 (64)	0	
Sulfamethoxazole		6 (24)	0	
Gentamicin		2 (8)	0	
Kanamycin		2 (8)	0	
Streptomycin		14 (56)	0	
Nalidixic acid		1 (4)	0	
Resistance to ≥3 antimicrobial agents		12 (48)	2 (10)	
Integron and antimicrobial resistance genes				
*intI*1		9 (36)	0	
*sul1*		3 (12)	0	
*aadA1*		3 (12)	0	
*bla*_TEM_		6 (24)	0	

*All isolates, regardless of geographic locale, were susceptible to ceftiofur, ceftriaxone, imipenem, amikacin, apramycin, chloramphenicol, ciprofloxacin, and trimethoprim/sulfamethoxazole. †No gram-negative bacteria were isolated from geese captured at Stone Mountain. ‡Isolates were susceptible to all 18 antimicrobial agents tested.

All *E. coli* isolates, except those from Craven County, were negative for class 1 integrons. Forty-four percent of *E. coli* isolates (n = 25) from Craven County Canada Geese possessed ≥1 antimicrobial-resistant determinant. Nine *E. coli* isolates were positive for class 1 integrase gene *intI*1; 6 isolates possessed a TEM β-lactamase.

## Conclusions

Outbreaks of illness associated with raw food products have been increasing, in part because of increased human consumption of fresh produce (1). However, several sources of preharvest contamination have been identified, including fecal material, contaminated irrigation water, and wild fowl (1). In a previous study, 32% of the *Salmonella* isolates from wild birds submitted to the Southeastern Cooperative Wildlife Disease Study were resistant to sulfamethoxazole, and 18.1% were resistant to both sulfamethoxazole and streptomycin (8). These findings are likely a result of interaction of these populations with environmental sources of enteric bacteria. In our study, the spectrum of *E. coli* resistance was very different among agricultural habitat geese, depending upon their exposure to livestock wastes. With growing populations of Canada Geese and associated evidence that they contribute to microbial water contamination (5,6), we hypothesized that observed resistance patterns might be related to the anthropogenic land usage of the bird habitats and that Canada Geese could serve as a vector of antimicrobial resistance genes between sources of fecal wastes and other environmental media. Little or no resistance was observed among the *E. coli* isolates recovered from Canada Geese in regions with no known direct contact with liquid wastes. However, geese in direct contact with liquid swine wastes had a significantly higher prevalence of antimicrobial resistance. Comparing these data with those reported recently by NARMS shows similar resistance profiles between *E. coli* isolates recovered from Canada Geese in contact with livestock wastes (Craven County) and those recovered from both food animals and fresh fruits and vegetables (12,13). In addition, a substantial number of isolates from several Canada Geese that had direct contact with lagoons containing liquid swine waste carried integrons and their associated resistance genes.

This and other studies suggest that resident, free-living, and migratory birds can be potential vectors of zoonotic pathogens, including antimicrobial-resistant variants, between waste-handling facilities and other agricultural resources, such as crops and water. Although all of our study populations of Canada Geese were nonmigratory, this species could serve to disperse bacteria between widely separated locations. In addition, since these birds use farm ponds and waste lagoons and graze on pastures inhabited by cattle and other livestock, the opportunities exist for new health problems in wildlife populations to emerge as well as new reservoirs of zoonotic disease to form. This work is the basis of continuing efforts to examine the potential role of wildlife in agricultural habitats as vectors of antimicrobial resistance in the environment.

This work was supported by USDA NRICGP grants 99-35212-8680 and 01-35212-10877 (Georgia) and USDA NRI Grant 99-35102-8178, USEPA IAG OD-5555-NTEX, and NIEHS training grant #ES07018 (North Carolina).
